# How Can Elispot Add Information to Improve Knowledge on Tropical Diseases?

**DOI:** 10.3390/cells6040031

**Published:** 2017-09-29

**Authors:** Josué da Costa Lima-Junior, Fernanda Nazaré Morgado, Fátima Conceição-Silva

**Affiliations:** 1 Laboratório de Imunoparasitologia, Instituto Oswaldo Cruz/FIOCRUZ, Pavilhão 26–4° andar, sala 406-C, Av. Brasil 4365, Manguinhos, 21045-900 Rio de Janeiro, Brazil; josue@ioc.fiocruz.br; 2 Laboratório de Pesquisa em Leishmaniose, Instituto Oswaldo Cruz/FIOCRUZ, Pavilhão 26–5° andar, sala 509, Av. Brasil 4365, Manguinhos, 21045-900 Rio de Janeiro, Brazil; morgado@ioc.fiocruz.br

**Keywords:** Elispot, infectious diseases, review, mycosis, protozoa, bacteria, vaccine, diagnosis, helminths

## Abstract

Elispot has been used as an important tool for detecting immune cells’ products and functions and has facilitated the understanding of host-pathogen interaction. Despite the incredible diversity of possibilities, two main approaches have been developed: the immunopathogenesis and diagnosis/prognosis of infectious diseases as well as cancer research. Much has been described on the topics of allergy, autoimmune diseases, and HIV-Aids, however, Elispot can also be applied to other infectious diseases, mainly leishmaniasis, malaria, some viruses, helminths and mycosis usually classified as tropical diseases. The comprehension of the function, concentration and diversity of the immune response in the infectious disease is pointed out as crucial to the development of infection or disease in humans and animals. In this review we will describe the knowledge already obtained using Elispot as a method for accessing the profile of immune response as well as the recent advances in information about host-pathogen interaction in order to better understand the clinical outcome of a group of tropical and neglected diseases.

## 1. Introduction

Scientific knowledge is always changing through the development of additional information and methods. Much is already known, but also much is still misunderstood. Therefore, in the biomedical field, new approaches to clarify the mechanisms involved in the balance of the function of cells, organs and systems are necessary to improve health maintenance. One of the most intriguing points is cells’ plasticity and their ability to adapt to needs. The immune system is no different, and as the cells involved in the inflammatory response can exhibit multiple functions, the body needs to cope with potential disequilibrium of organs and systems that trigger disease, driving different immune responses. In this context, and with the advance of biotechnology, many new methodologies can be used to better describe the type and intensity of the immune response. Why is this particularity so important? In the book “Germ Warfare: breakthroughs in immunology,” the author recounts a conversation that took place in 1953 between two researchers about a sentence from Robert Rich, an American pathologist who said, “Lymphocytes can be always being observed after a day or two in areas of inflammation ... but there is not the slightest notion of what they are doing there ... Literally, nothing of importance is known regarding the potentialities of these cells ...” In scientific terms, it has not been that long, but today it is impossible to conceive of the immune response without the central and important action of lymphocytes.

**Figure 1:**
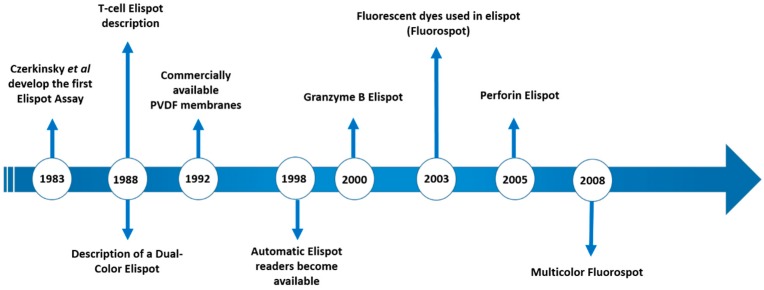
Timeline of the main Elispot advances over the past 3 decades.

**Figure 2:**
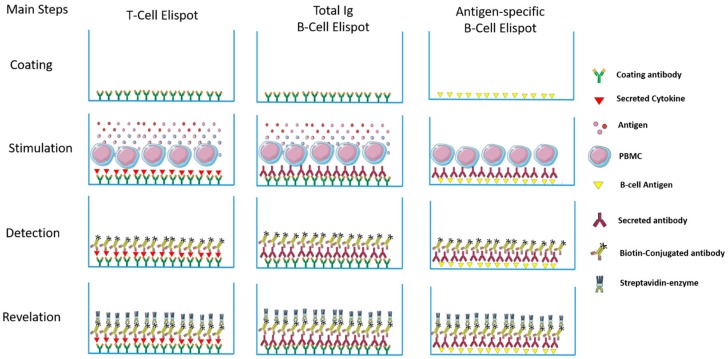
Schematic representation of the main steps on different Elispot categories used in the Tropical diseases field. Briefly, in the T-cell Elispot, a specific antibody is captured for the chosen Interleukin and is coated onto a PVDF plate and incubated, generally, overnight. After the blocking step, cells are added along with an appropriate stimulant (peptide antigen/positive control/polyclonal activator) and incubated for 12–96 h according to the cytokine under investigation. Therefore, secreted cytokines are captured by the immobilized antibody on the PVDF surface. After washing, a biotinylated detection antibody is added to allow detection of the captured cytokine and the secreted cytokine is visualized using an avidin-HRP or avidin-ALP conjugate and a colored precipitating substrate. In the total Immunoglubulin B-cell Elispot, the steps are the same for the detection of the secreted antibody. However, for antigen-specific B-cell quantification, the plates are coated overnight with the target antigen and after a blocking step, cells are incubated for 12 h–96 h. The specific immunoglobulins that are secreted bind the antigen. Then, the antigen-specific antibodies are detected by biotinylated anti-IgG and the revelation of the spots generated is achieved by the same procedure as others.

Th1 and Th2 subsets of T lymphocytes were first described 30 years ago [1,2] and the immune response was divided into pro-inflammatory (type 1) and anti-inflammatory (type 2) responses. Since then, an incredible number of subsets, new functions, cooperation, regulation etc. have been described [3,4,5]. Thus, a better understanding of the mechanisms involved in the induction of the inflammatory reaction/immune response is important. When we think about infectious diseases, the identification of the predominant type of response may aid understanding of the evolution of infections in a mammalian host. Consequently, this can lead to the development of new intervention tools to avoid or control pathogens, as vaccines and drugs respectively. To evaluate the profile of different types of responses, a variety of supernatant-based and cell-based methodologies can be employed as a lymphoproliferative response—ELISA, Multiplex, flow cytometry, Elispot, and comparisons between them have been the subject of different studies [6,7,8]. Among them, Elispot has the potential to quantitatively verify the distribution of cells responding to a given stimulus and to characterize the type of predominant response, and has been used to detect a variety of immune profiles [9,10,11]. The first description of the Elispot assay was published over 30 years ago and has been constantly upgraded over time (Figure 1). Despite the Elispot assay originally being developed to detect antigen-specific antibody-secreting B cells before being evolved for the enumeration of antigen-specific cytokine-secreting T cells, many other improvements were made to assay techniques and reagents (Fluorescent dyes, dual-labeling and others) and allowed a wide range of utilities for the detection of specific B and T cellular responses at the single cell level, using a simple methodology (Figure 2). 

**Figure 3:**
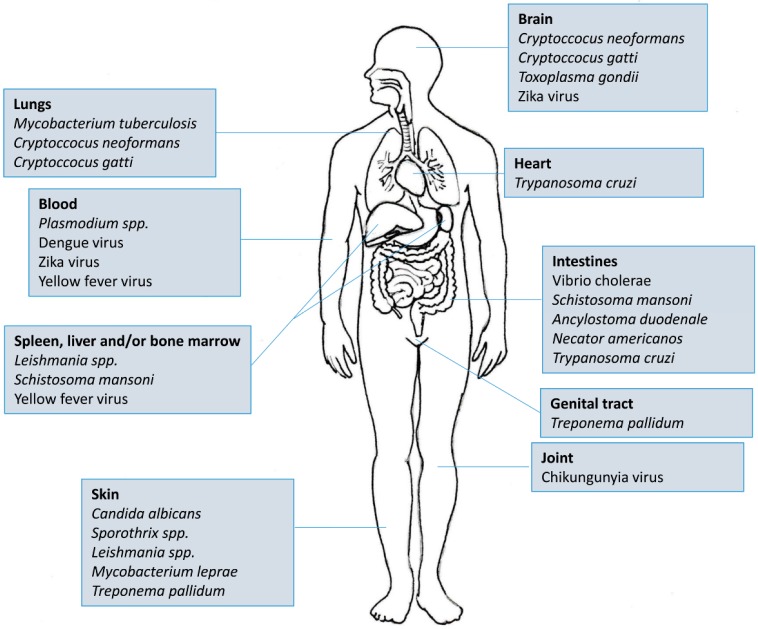
Etiological agents of tropical and/or neglected diseases in relation to major affected areas of the body.

There are more than 5600 indexed articles that use Elispot as a tool for medical research. Of this total, almost 3000 are directed towards four main diseases/areas: Cancer (n = 1309), HIV (n = 944), Allergy (n = 359) and Autoimmunity (n = 245). If we take into account that tropical and neglected diseases (Figure 3) affect more than half of the world’s population and most of them also stimulate cellular and antibody responses, it becomes clear that Elispot is a powerful and straightforward tool for understanding the mechanisms underlying these pathogens. Moreover, as the plates can be stored at room temperature for future analysis, the use of Elispot in tropical endemic settings is more feasible than other techniques such as Flow cytometry, Real-Time PCR or Cellular proliferation. Therefore, in this review, we have focused on the employment of Elispot to measure some aspects of the immune response in different tropical infectious diseases caused by viruses, bacteria, protozoans, fungi and helminths.

## 2. Intracellular Protozoa: *Plasmodium* spp., *Toxoplasma gondii, Leishmania* spp. and *Trypanosoma cruzi*

**Figure 4:**
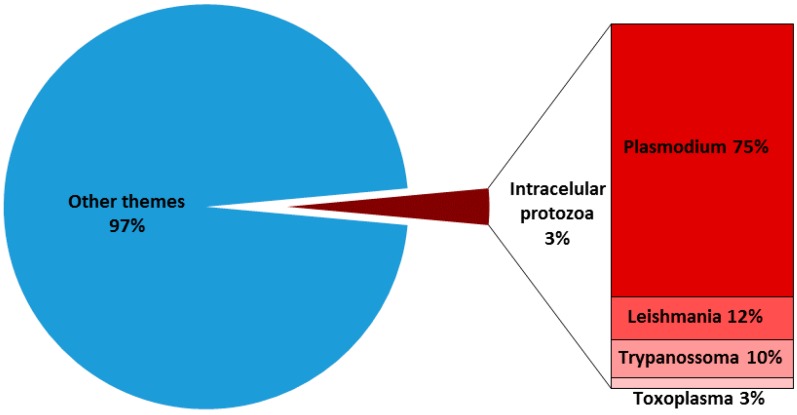
Prevalence of Elispot indexed (PubMed) articles in tropical diseases caused by intracellular protozoa in relation to overall Elispot articles published until June 2017.

T-cells are critical for defense against protozoan parasites. In general, the development of a Th1 cell response is required since the production of IFN-γ, TNF is critical for the activation of phagocytes, as well as for the expression of cytokines, and cell surface molecules are needed to generate effector CD8^+^ T cells that can recognize and kill infected host cells. Over the past few years, much has been learnt about the molecular and cellular components necessary for the generation of T cell responses, and it has become clear that these responses need to be tightly controlled during pathogenesis and also in developing tools for disease prevention. In all these cases, Elispot assay can be particularly helpful. However, despite the widespread and increasing use of Elispot in protozoan parasite research, there are fewer than 4% of published articles that use Elispot (Figure 4). In this scenario, only four intracellular parasites were the main target in Elispot applications over the past few years: *Plasmodium* spp., *Toxoplasma gondii*, *Leishmania* spp. and *Trypanosoma cruzi*.

The *Plasmodium* genus belongs to the Apicomplexa phylum, and this parasite has a complex life cycle. Allied to the highest burden and mortality in tropical and sub-tropical parts of the globe, the different forms and targeted cells during human infection make the access to the immune response and pathogenesis by Elispot particularly wider than that of other protozoan parasites [12,13,14]. In vaccine development, which represents the major application of Elispot in malaria, several papers have already reported the ex-vivo responses of exposed and/or vaccinated individuals against synthetic peptides representing T-CD8 [15,16,17,18,19,20] and T-CD4 [17,21,22] epitopes in order to identify vaccine candidates. Moreover, the validation of HLA-restricted [15] or promiscuous epitopes [23] was also largely accessed by measuring IFN-γ and IL-4 responses using Elispot. In relation to humoral response, antibodies still constitute a critical component of the naturally acquired immunity that develops following frequent exposure to malaria. However, specific antibody titers have been reported to decline rapidly in the absence of reinfection, supporting the widely perceived notion that malaria infections fail to induce durable immunological memory responses, which also makes vaccine development the great challenge within the vaccinology field. 

More recently, with the advantage of a large variety of commercially available kits for B-cell Elispot, the longevity of both antibody and B cell memory responses to malaria antigens among individuals who were living in transmission areas has been assessed by this approach [24]. On the other hand, the use of Elispot as a tool for identifying cells and mediators of innate immunity is restricted to the identification of NK cell secreting granzyme B in naïve and exposed volunteers [25]. Actually, there is no Elispot approach able to predict current infections, pathogenesis and/or clinical complications, but Walker and colleagues (2015), in experimental human malaria infection, tried to correlate a Malaria-specific T-cell in the form of IFN-γ and IL-4 with parasitemia but their magnitude did not correlate with the parasite load [26]. Moreover, the frequency of T-cell responses obtained by the standard Elispot assay, quantifying effector memory T cells, does not correlate well with disease control or protection with some vaccine candidates. In fact, the use of Elispot in malaria research—besides in the vaccine field—needs to be further explored in order to find more conclusive associations.

Another parasite from Apicomplexa, *Toxoplasma gondii* is an intracellular coccidian protozoan. In various places throughout the world, it has been shown that up to 95% of some populations have been infected with Toxoplasma. Although quite common in tropical and sub-tropical regions, manifesting in a sub-clinical or asymptomatic manner, the infection can be serious in certain situations (pregnancy and HIV/AIDS). Regarding the immune response, *T. gondii* infection stimulates a strong and persistent response mediated by T-CD4 and T-CD8 cells, characterized by the production of proinflammatory cytokines including IL-12, IFN-γ and TNF, which contribute to the intracellular destruction of the parasite [27]. Despite this varied profile of the immune response, the use of Elispot to evaluate the naturally acquired immune response against T. gondii antigens is practically absent, even if currently there is no vaccine for toxoplasmosis. The use of Elispot in toxoplasmosis is basically restricted to only two works on the evaluation of cellular response aimed at the screening of vaccine candidate epitopes [28,29]. In both cases, IFN-γ Elispot was used to measure the number of antigen-specific T-cell responses. Regarding the diagnosis or prognosis of toxoplasmosis using Elispot as a tool, there is no report in the literature. However, since toxoplasmic encephalitis (TE) is one of the most important opportunistic infections of the central nervous system in patients infected with human immunodeficiency virus (HIV)-1, Hoffman et al. evaluated, using IFN-γ-specific Elispot tests, the effect of highly active anti-retroviral therapy (HAART) and *Toxoplasma gondii*-specific immune responses on the occurrence of TE [30]. Interestingly, those patients with an acute episode of TE or a TE relapse had a significantly lower frequency of *T. gondii*-specific activated T-cells than patients who discontinued the maintenance therapy and were relapse-free. This data suggests that the evaluation of *T. gondii*-specific immune responses by Elispot can improve the estimates of the individual risk of TE and TE relapses.

*Trypanosoma cruzi* is the etiologic agent of Chagas disease, a neglected tropical disease that affects millions of people mainly in Latin America. Upon infection, a strong immune response is triggered, which has both protective and pathological consequences. During this process, T-cells and their mediators are largely stimulated. Therefore, in addition to epitope mapping and vaccine development [31,32,33,34,35], the knowledge of *T. cruzi*-specific immune responses might serve as surrogate indicators of treatment success. Taking these particular features into account, Alvarez and colleagues addressed—in a long-term follow-up study—whether the cure achieved after treatment can be predicted by changes in anti-parasite antibody response by serological tests and the frequency of specific T-cell response by Elispot [36]. Interestingly, changes in the functional status and potential of *T. cruzi*-specific T cells, indicative of reduced antigen stimulation, provided evidence of cure following the treatment. This finding was fundamental to not only evidence that the cure was associated with an early decline in *T. cruzi*-specific IFN-γ-producing T cells, but also highlighted the importance of the Elispot approach in the follow-up of patients infected with intracellular protozoa.

Parasites belonging to the *Leishmania* genus are etiological agents of cutaneous, mucocutaneous, and visceral diseases in humans and mammals. Leishmaniasis comprises one of the diseases included in WHO programs for control and elimination of neglected tropical diseases [37,38] and 0.7 to 1.3 million of new cases of cutaneous leishmaniasis are estimated worldwide each year. The immune response against *Leishmania* spp. presents a spectrum range from high levels of cell-mediated immunity to high levels of antibody. Although all clinical forms require Th1 responses to cure the disease, an exacerbated response and an increased number of CD8 cytotoxic T-cells are also associated with increased disease severity. The consequence of an extremely exaggerated cellular response is the development of mucosal leishmaniasis, in which parasites metastasize to the nasopharyngeal mucosa and cause disfiguring lesions. On the contrary, patients at the other end of the spectrum have high parasite numbers within the lesions, which is a consequence of low levels of Th1 cytokines. Despite this rich and variable scenario for Elispot applications in the quantification of antigen-specific T-cells and clinical outcome, the use of Elispot is still more biased to the identification and validation of several T-CD8 epitopes aimed at vaccine design [33,39,40,41], follow-up after pre-clinical studies [42], and selection of HLA restricted epitopes [39]. Only recently, the frequency of IFN-γ and IL-10 specific cells has been accessed in order to follow up patients presenting worsening cutaneous ulcers during pregnancy. In this case report study, antigen-specific cytokine secretion by PBMC was assessed by Elispot assay at 8 months of pregnancy and 2–6 months after birth. IFN-γ secretion was decreased in patients during pregnancy but increased postpartum and antigen-specific IL-10–producing cells increased in response to specific re-stimulation during pregnancy [43]. In relation to the humoral immune response against *Leishmania* spp, a combination of immunomagnetic cell sorting and Elispot techniques was used to permit the enrichment and characterization of antibody-secreting cells (ASC). Cell suspensions containing putative ASC were sorted and the resulting cell populations were examined for the presence of ASC by Elispot assay. As a model system, the expression of selected cell differentiation markers by human circulating ASC has been evaluated after parenteral tetanus vaccination and during the course of a Leishmania infection [44]. Even if the model could be particularly useful in situations where ASC are present at low frequencies, it has not yet been replicated using large cohort samples of *Leishmania* spp infected individuals.

## 3. Tropical Arboviruses

Dengue fever is one of the most neglected tropical diseases and is of the highest international public health importance, with 50 million cases worldwide every year [45]. It is caused by four antigenically distinct serotypes of dengue virus (DENV1–4), and although serotype-specific and strongly neutralizing cross-reactive immune responses against the four DENV serotypes are thought to be protective, subneutralizing Abs can contribute to increased disease severity upon secondary infection with a different DENV serotype [46].

Dengue is the most studied arbovirus evaluated by Elispot for B or T-cells detections. Activation of dengue serotype-cross-reactive memory CTL during secondary dengue virus (DENV) infection is thought to be important in the pathogenesis of dengue hemorrhagic fever. T lymphocyte responses in these viral infections might also be modified by partial sequence homology and an altered peptide ligand effect [47]. In 2001, Loke et al. observed an increase in the frequency of dengue virus epitope - specific CD8 T-cells secreting IFN-γ in patients presenting dengue hemorrhagic fever supporting both the protective and pathogenic roles of these cells [48]. An early CD8 T-cell secreting IFN-γ in response to dengue virus epitopes in adult patients with secondary dengue virus infection was detected highlighting the importance of nonstructural 3 (NS3) peptide and cross-reactive T cells during acute secondary infection [49,50]. Using an ex vivo IFN-γ Elispot assay, Malavige et al. (2012) observed that patients with severe dengue had a lower total number of T cells. The NS3 specific T cells persisted and produced high levels of IFN-γ but not TNF-α, IL-3, IL-13, IL-2, IL-10 or IL-17. Subsequently, the authors suggested the contribution of IL-10 to the pathogenesis of acute dengue infection by inhibiting DENV-specific T cell responses, which can be restored by blocking IL-10 [51].

Jeewandara et al. (2015) studied the functionality of DENV specific memory T cell responses in relation to clinical disease severity [52]. Using ex vivo IFN-γ Elispot assays, they investigated the functionality of DENV-specific memory T cell responses in 338 individuals from Sri Lanka, who were naturally infected and were either hospitalized due to dengue or had mild or sub clinical dengue infection. The results suggested that the T cells of individuals with both past mild or sub clinical dengue infection and who were hospitalized produced multiple cytokines when stimulated with DENV-NS3 peptides. However, while DENV-NS3 specific T cells of those with mild/sub clinical dengue infection were more likely to produce only granzyme B, those who were hospitalized were more likely to produce both TNF-α and IFN-γ or TNF-α alone [52].

Invariant natural killer T (iNKT) cells have been shown to play a significant role in many viral infections and were observed to be highly activated in patients with acute dengue infection. They are capable of rapid activation and production of cytokines upon recognition of antigenic lipids presented by CD1d molecules. Kamaladasa et al. (2016) investigated the proportion of iNKT cells and their phenotype in adult patients with acute dengue infection by both IFN-γ and IL-4 ex-vivo Elispot assays following stimulation with alpha-galactosyl-ceramide (aGalCer) and suggested that in acute dengue infection there is an expansion of highly activated CD41 iNKT cells [53].

Quantitation of the antigen-specific memory B cell response after vaccination or infection may be an important and independent measure of long-lived immunity and the amplitude of recall serum antibody titers [54]. The precise measurement and isolation of B memory cells is technically challenging due to their low frequencies in the circulating blood. Memory B cell populations in human peripheral blood are extremely small, and due to the high sensitivity of Elispot this technique becomes the tool of choice for analyses of low frequency cells. 

B cell response in primary and secondary dengue infections has also been evaluated by Elispot and specific antibody detection is important in vaccine and immunopathogenesis studies. The method described by Mathew et al. (2011) used DENV E or NS1 proteins coated plates and the cells from patients were incubated [55]. The detection of specific IgG was realized using anti-human IgG. Total IgG was detected in plates coated with anti-human IgG. The authors suggested that the minor populations of serotype-cross-reactive B cells generated by primary DENV infection are preferentially expanded during secondary DENV infection. Friberg et al. (2012) showed that anti-DENV antibodies are cross-reactive and directed against multiple proteins [56].

Understanding the breadth of the immune response in natural DENV infections and in vaccines is crucial for determining the correlates of protection or disease severity [46]. Potential epitopes for DENV vaccination have been evaluated using Elispot assay. In a computational prediction study four dengue virus-specific CD4 T-cell epitopes were identified and the frequency of IFN-γ producing cells was measured by Elispot [57]. Subsequently, many other authors used the same methodology to identify candidate epitope-based vaccines [58,59,60,61,62,63]. In order to study the immunogenicity of candidate vaccines in humans, a dendritic cell-based IFN-γ Elispot assay using E protein-pulsed dendritic cells as antigen presenting cells and non-adherent cells freshly separated from whole PBMCs as the source of T cells was developed [64]. They suggested that the dendritic cell-based IFN-γ assay is a useful tool for assessing immunological memory for clinical research. Weiskopf et al. (2015) used IFN-γ Elispot assay to evaluate the human CD8^+^ T cell responses induced by a live attenuated tetravalent dengue vaccine directed against highly conserved epitopes [65]. In the same manner, Simmons et al (2016) evaluated recombinant dengue 2 virus NS3 helicase protein that enhanced antibody and T-cell response of a purified inactivated vaccine [49].

It can also be used as a microneutralization plaque assay as described by several authors [66,67,68,69,70,71]. Liu et al. (2001) compared a plaque reduction neutralization assay (PRNT) with a microneutralization enzyme-linked immunospot-based assay (Elispot-MNT) to measure the neutralizing activities of antibodies specific to dengue virus envelope protein and they pointed some advantages for Elispot-MNT: 1- Elispot-MNT is faster than PRNT, has a higher throughput, and is more objective [69]. In addition, PRNT must be performed in six-well plates and requires a large volume of immune serum, potentially imposing a limitation on experiments with low-titer sera, and the fact that clinical DENV isolates do not form clear plaques in cultured cell monolayers highlights the difficulty in using PRNT to detect DENVs [66]. 

Early detection of Dengue infection can decrease mortality rates from above 20% to less than 1% and the relevant early diagnosis analyte is the viral non-structural glycoprotein, NS1 [45]. In this context, recent advances have been made in diagnosis of Dengue based on Elispot assay using serum from patients. Linares et al. (2013) developed an immunospot assay using fluorescent nanoparticles for dengue fever detection. They described an easy method combining principles of fluorophore linked immunosorbent assay (FLISA) and enzyme linked immunospotting (Elispot) using mouse anti-NS1 IgG labeled with fluorescent nanoparticles. The use of fluorescent nanoparticles provides higher sensitivity than an assay using usual fluorescent dye molecules, besides avoiding bleaching effects and proves to be a suitable method for Dengue NS1 detection in impoverished regions or epidemic areas. With the same objective, Hadjilaou et al. (2015) modified the conventional Elispot to develop a Quad-Color FluoroSpot to provide a means of examining B cell/Ab serotype specificity and cross-reactivity on a single-cell basis [46]. Abs secreted by B cells are captured by an Fc-specific Ab on a filter plate. Subsequently, standardized concentrations of all four DENV serotypes are added to allow equal stoichiometry for Ag binding. After washing, the spots, representing individual B cells, are visualized using four fluorescently labeled DENV serotype-specific detection mAbs. This method can be used to better understand the breadth and magnitude of B cell responses following primary and secondary DENV infection or vaccination and their role as immune correlates of protection from subsequent DENV infections.

There is only one paper evaluating Zika virus epitopes using the Elispot assay [72]. The authors identified 25 HLAB * 0702 restricted epitopes and 1 HLAA * 0101 restricted epitope and intracellular cytokine staining (ICS) in ZIKV infected IFNα/β receptor deficient HLA transgenic mice. The cross-reactivity of ZIKV epitopes to dengue virus (DENV) was tested using IFN-γ Elispot and IFN-γ ICS on CD8 T cells from DENV infected mice, and five cross-reactive HLAB * 0702 binding peptides were identified by both assays. ZIKV/DENV cross-reactive CD8 T cells in DENV immune mice expanded post ZIKV challenge and dominated in the subsequent CD8 T cell response. ZIKV challenge following the immunization of mice with ZIKV specific and ZIKV/DENV Cross-reactive epitopes elicited CD8 T-cell responses that reduced infectious ZIKV levels, and CD8 T cell depletions confirmed that CD8 T cells mediated this protection. These results identified ZIKV specific and ZIKV/DENV cross-reactive epitopes and demonstrate both an altered immunodominance pattern in the DENV immune setting relative to naive, as well as a protective role for epitope specific CD8 T cells against ZIKV.

Chikungunya virus (CHIKV) is an alphavirus that causes chronic and incapacitating arthralgia in humans. Injury to the joint is believed to occur because of viral and host immune-mediated effects [73]. A previous study using IFN-ƴ Elispot assay indicated that CHIKV-specific CD4—but not CD8^+^—T cells were essential for the development of joint swelling without any effect on virus replication and dissemination. Infection in IFN-γ^−/−^ mice demonstrated that pathogenic CD4 T-cells do not mediate inflammation via an IFN-γ–mediated pathway. These observations indicate that mechanisms of joint pathology induced by CHIKV in mice resemble those in humans and differ from infections caused by other arthritogenic viruses, such as Ross River virus.

To characterize the immunity developed by patients infected by CHIKV, Hoarau et al. (2013) studied the intensity and specificity of CHIKV-specific T cells mediated responses in chronic and recovered patients at 12 to 24-month post-infection [74]. T cells producing IFN-γ were detected against CHIKV in 85% patients’ cells either by direct Elispot assay (69% patients) or after expansion of memory T cells allowing the detection of both CD4 and CD8 specific-T cells in 16% additional cases. The IFN-γ response was mainly engaged in response to nsP1 or E2 (52% and 46% cases, respectively) but in only 27% of cases against the capsid. The anti-E2 response represented half the magnitude of the total CHIKV IFN-γ production and was mainly directed against the C-terminal half part of the protein. Almost all patients had conserved a T cell specific response against CHIKV with a clear hierarchy of T cell responses (CD8 > CD4) engaged against E2 > nsP1 > capsid. Most importantly, the intensity of responses was not significantly different between recovered and chronic patients. These findings constitute key elements to a better understanding of patient T cell immunoreactivity against CHIKV and argue against a possible defect of T cell immunoresponse in the chronicity post-CHIKV infection.

Yellow fever virus (YFV) is a reemerging problem despite the existence of an effective live-attenuated vaccine. The induction of YFV-neutralizing antibodies undoubtedly contributes to vaccine efficacy, but T lymphocyte responses to YFV likely play a role in long-term efficacy [75]. T lymphocyte responses to YFV in four vaccines were studied and 13 YFV-specific CD8- CTL lines that recognized epitopes on the E, NS1, NS2b, and NS3 proteins; eight CTL lines were HLA-B35-restricted. YFV-specific T cell responses were detectable by IFN- Elispot assays 14 days after vaccination, with T cell frequencies sustained for up to 19 months. The authors indicated that the live 17D YFV vaccine-induced CD8-T cell responses directed against at least four different HLA-B35-restricted YFV epitopes. Immunity to YF is conferred by the interplay of humoral and cellular immune responses [76]. The analyses of Th1/Th2 cytokines by IFN-γ and IL-4-Elispot was performed in 12 healthy adults vaccinated with 17D YFV. IFN-γ and IL-4-producing cells were significantly increased on the 15th day after vaccination in all volunteers. According to the authors, these results suggest that cellular response and antibody production are important features of the response to 17D YFV vaccine. Recently, T cell responses were screened ex-vivo by IFN-ƴ Elispot assays using blood samples from 220 17D YFV vaccines collected from two months to four years after immunization [77]. 653 peptides covering the envelope (Env) and nonstructural (NS) proteins 1 to 5 were studied. The authors suggested that in addition to factors of the innate immunity, promiscuous T-cell antigens might contribute to the high efficacy of the yellow fever vaccines.

## 4. Bacterial Infections in the Tropics: *Mycobacterium tuberculosis, Mycobacterium leprae, Treponema pallidum, Vibrio cholerae*

Bacterial infections are common in tropical parts of the world and can include those species also regularly seen in temperate climates. Generally, bacterial infections in the tropics include bartonellosis, yaws, pinta, melioidosis, glanders and others. However, the bacterial diseases that have the most severe medical and economic impact in low- and middle-income countries are those caused by members of the *Mycobacterium* genus, and millions of persons throughout the world suffer from tuberculosis and leprosy. Coincidently, in this scenario, Elispot has emerged as a powerful tool in several fields related to mycobacterium research and the main applications are directed towards the diagnosis, vaccine development and evaluation of clinical outcome.

Tuberculosis (TB) is an airborne infectious disease caused by organisms of the *Mycobacterium tuberculosis* complex. Although primarily a pulmonary pathogen, *M. tuberculosis* can cause disease in almost any part of the body. Infection with *M. tuberculosis* can evolve from containment in the host—in which the bacteria are isolated within granulomas (latent TB)—to a contagious state, in which the patient will show symptoms that can include cough, fever, night sweats and weight loss. In many low-income and middle-income countries, TB continues to be one of the main causes of morbidity and mortality [78]. The immune response against *M. tuberculosis* is mainly biased for CD4 T-cells, as well as the cytokines IL-12, IFN-γ, and TNF, which are critical in the control of Mycobacterium tuberculosis infection. Taking into account this large burden and the biased cell-mediated immune response, it is not a surprise that the use of Elispot in TB research began very quickly after the technique description in the middle 1980s.

**Figure 5:**
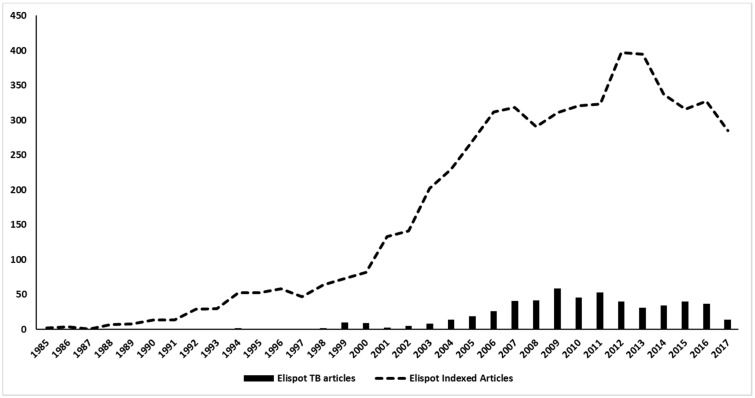
Number of PubMed indexed articles using Elispot technique in the Tuberculosis field since the first description in 1990.

The first report of Elispot on TB research was done by Lu and colleagues (1990), which used a “nitrocellulose immunospot assay” to enumerate cells secreting anti-BCG IgG and IgM antibodies in order to assess the diagnosis of tuberculous meningitis [79]. Surprisingly, the detection of anti-BCG IgG antibody-secreting cells presented a higher sensitivity and specificity than ELISA, and represented a useful method for complementing the early diagnosis of tuberculous meningitis. After this first approach, Elispot applications in the TB field increased more than in many other diseases, being used in more than 500 articles on tuberculosis over the years (Figure 5). Actually, the publications with Elispot and TB represent nearly 10% of all Elispot published articles in history. Most of these articles are in the diagnosis field, which is actually the main focus of Elispot research on TB.

One of the reasons for this observation is the wide use of IFN-γ-based assays, collectively known as IFN-γ release assays (IGRAs), which have emerged as a reliable alternative to the old tuberculin skin test (TST) for the immunodiagnosis of TB infection. Commercially available tests, including the ELISA, QuantiFERON-TB (QFT-IT) and the Elispot based T-SPOT. TB are more accurate than TST for the diagnosis of TB, since they are highly specific and correlate better with the existence of risk factors for the infection [80,81]. Moreover, according to the available data, the T-SPOT.TB presented higher number of positive results than QFT-IT. In addition, data from meta-analysis studies suggest that IFN-γ-based tests are better predictors of the subsequent development of active TB than TST [82]. Recent publications also suggest that the accuracy in discriminating LTBI from active tuberculosis can be improved by parallel assessment of the secreting profile of T-cells for other cytokines, such as IL-2 and TNF-α [83,84,85].

Nevertheless, the clinical usefulness of this approach still needs to be investigated and this has so far mainly been addressed using flow-cytometry that still represents the technical gold standard for multiparameter analysis of immune cells [86]. However, flow cytometry analysis is an expensive and labour-intensive technique to be applied in low- and middle-income countries. Meanwhile, the two color FluoroSpot enables to simultaneously assess individual cells that produce cytokines IFN-γ and IL-2 after antigen stimulation and can be used. Chesov and colleagues evaluated whether dual (IFN-γ and IL-2) cytokine profile analysis by FluoroSpot is superior to the single Elispot-IGRA technology to distinguish between different states of *M. tuberculosis* infection [87]. Although antigen-specific IL-2- IFN-γ+ secreting T-cells are elevated in active tuberculosis in comparison to past tuberculosis and LTBI and can be easily identified by FluoroSpot, the parallel diagnosis of IL-2 and IFN-γ secretion by antigen-specific T-cells does not allow a reliable differentiation between different states of *M. tuberculosis* infection in clinical practice. In fact, the application of Elispot in TB diagnosis has very good potential but more approaches are still needed to clearly distinguish active and latent TB.

In relation to vaccine development, despite the variability in its efficacy, the BCG vaccine has proven that protective immunity against TB can be induced by a vaccine, even though the protective mechanism is not well elucidated. The development of TB vaccines faces numerous challenges and at least 13 vaccine candidates are currently being tested on different platforms (whole-cell or lysates of mycobacteria, viral vector vaccines and adjuvanted recombinant protein vaccines) [78]. To date, tuberculosis (TB) vaccine candidates have been designed predominantly to stimulate a T helper 1-type CD4 T-cell response, and as a natural consequence Elispot has been used in most of them in order to access the specific cellular response. The viral vector candidates alone or in combination typically stimulate a CD8 T-cell response. The whole-cell and lysate mycobacterium-based candidates have the greatest potential to stimulate other aspects of the host innate and adaptive immune system, including, for example, donor unrestricted T cells (such as γδ-cells, mucosal-associated invariant T-cells, CD1-restricted T-cells and natural killer T-cells), as they present the broadest array of antigens.

Interestingly, despite the large contribution of Elispot in the *M. tuberculosis* field, little contribution is described for *M. Leprae*. The first description appeared as late as 1996, when Kifayet and colleagues described for the first time the frequencies of antibody-secreting cells in Erythema nodosum leprosum (ENL), a serious complication of lepromatous (L) disease [88]. However, the authors found no correlation between serum concentrations of IgG antibodies and the frequency of antibody producing cells. In relation to T-cell specific response, the few studies that use Elispot describe a cross-reactivity between *M. leprae* and *M. tuberculosis* ESAT-6 and culture filtrate protein-10 (CFP-10) antigens. In both antigens Elispot was used to demonstrat that it is a highly potent T-cell-stimulating antigen. However, there was clear evidence of cross-reaction, which has several implications for the use of ESAT-6 and CFP-10 as diagnostic tools in TB and leprosy [89,90].

Yaws is a tropical infection of the skin, bones and joints caused by the spirochete bacterium *Treponema pallidum*. Specific serologic assays for syphilis cannot differentiate current infections from past infections and are inefficient for monitoring the efficacy of antibiotic therapy. In the late 1990s, enzyme linked immunospot assays were developed in an attempt to differentiate active syphilis from treated syphilis [91]. In these assays, blood mononuclear cells are collected and tested for *T. pallidum*-specific circulating antibody-secreting cells by Elispot. An early work showed that they were positive in 100% of patients with primary syphilis, 87% of patients with secondary syphilis, and 46% of patients with latent syphilis. However, there is no mention of this test in later papers or in current recommendations for syphilis diagnosis. Its major use in syphilis is to distinguish active infection in the newborn from passive transfer of antibodies from the mother to the infant [92]. Stoll et al. found that sensitivities of the “reverse Elispot” were not as high as the IgM ELISA or FTA-abs but its specificity was >96%.

Cholera is a diarrheal disease caused by infection with *Vibrio cholerae*, a bacterium most often found in contaminated water and shellfish, which produces a toxin that upsets the biochemical balance of cells lining the intestine and makes them secrete copious amounts of water and electrolytes. Infection with *Vibrio cholerae* induces durable immunity against subsequent disease, a process hypothesized to reflect anamnestic immune responses at the intestinal mucosa. The presence of antigen-specific memory B-cells may therefore be a more direct measure of protection than serum antibody responses. Therefore, the main application of Elispot in this disease is the enumeration of a specific B-cell and the evaluation of memory. Despite the few studies on this theme, Jayasekera et al. (2008) measured immunoglobulin (Ig) G memory B cells specific for cholera toxin B subunit (CTB) in patients up to 90 days after *V. cholerae* infection by Elispot [93]. Interestingly, CTB-specific IgG memory B cell responses were detectable in the circulation at least 3 months after infection and remained measurable even after serum antibody titers had declined to undetectable or considerably lower levels. Therefore, the data generated indicated B-cell Elispot for the detection of antigen-specific memory B-cells may be a better long-term marker exposition to *V. cholera* than the simple evaluation of circulating antibodies by Elisa [94].

## 5. Helminths

Helminths affect one third of the population worldwide. Schistosomiasis and soil-transmitted helminth infections are among the world’s most prevalent afflictions of humans who live in areas of poverty in the world. These infections give rise to much suffering and death. In addition, they contribute to the perpetuation of poverty by impairing the physical and intellectual growth of children, and by diminishing the work capacity and productivity of adults [95]. Elispot assay has been applied in helminthology to evaluate immunopathogenesis, comorbidity studies and the efficacy of vaccine candidates. In 1993, King et al. used Elispot to show that helminth-induced serum IgE levels are directly related to an increased capacity by PBMC to produce IL-4 and inversely associated with IFN-ƴ production [96].

Schistosomiasis is the most frequent helminthiasis evaluated by Elispot. The first study in *Schistosoma mansoni* infection using Elispot took place in 1994 [97]. Cytokine responses of peripheral blood mononuclear cells from humans infected with *S. mansoni* were assessed. Persons with acute and hepatosplenic infections produced higher levels of IL-4 and IL-5 and higher frequencies of IL-4 producing cells in response to mitogen than uninfected persons did. In contrast, mitogen-induced production of the Th1 cytokine IFN-ƴ did not differ from that of uninfected controls. Nevertheless, in cells stimulated with adult worm antigen, a more mixed Th0 type response was observed with the production of both Th1 and Th2 cytokines. These results support the conclusion that Schistosoma infection results in increased production of Th2 cytokines. Years later, the Elispot assay was used to prove that *Schistosoma mansoni* infection inhibits cellular immune responses to core HCV peptides showing a significant decrease in core-specific T-cell IFN-ƴ, IL-4 and IL-10 responses [98].

Patients co-infected with the hepatitis C virus (HCV) and the trematode *Schistosoma mansoni*, had an increased incidence of viral persistence and accelerated fibrosis due to this type 2 response. Subsequently, immune response and the protective efficacy of DNA vaccine formulation for Schistosomiasis (Sm-p80) were evaluated [99]. PBMCs produced appreciably more spot forming units for INF-γ than for IL-4 in Elispot. Overall it appears that even though a mixed (Th1/Th2) type of humoral antibody response was generated following immunization with Sm-p80; the dominant protective immune response is Th1 type. 

Recently, the effect of current *S.mansoni* infection on the immunogenicity of a candidate TB vaccine, MVA85A, in BCG vaccinated adolescents was taken into account in an open-label trial of phase II [100]. The primary outcome was immunogenicity measured by Ag85A-specific IFN-γ Elispot assay, which did not show differences between uninfected and *S. mansoni* infected volunteers, suggesting this vaccine was applicable and safe in this population. In 2014, the effect of maternal helminth infection on maternal and neonatal immune function and immunity to tuberculosis was evaluated [101]. *M. tuberculosis* and helminth infection each affect each, one third of the world population. Helminth infections down-regulate cell mediated immune responses and this may contribute to lowering the efficacy of BCG vaccination and to increasing the prevalence of tuberculosis. In this study, 85 pregnant women were screened and 23 presented helminthic infection. The most common parasite was *S. mansoni* detected in 20% of parasitized women. Maternal helminth infection had a significant association with the reduction of IFN-γ response of cord blood mononuclear cells, increased of total IgE and cross placental transfer of TB-specific IgG [101]. Interestingly, Abate et al. (2015) showed that asymptomatic helminth infection in active tuberculosis is associated with increased regulatory and Th-2 responses and a lower sputum smear positivity [102]. They used Elispot to measure IL-5, IL-10 and IFN-γ expressing cells. The authors discussed that anti-helminthic treatment in TB patients may, therefore decrease the worm burden, leading to the impairment of IL-10 production and improving cellular immunity to TB which could have potential clinical benefits.

The impact of helminthic infections in autoimmune diseases has also been analyzed using Elispot in two studies [103,104]. Helminths have been used to inhibit intestinal inflammation in patients with Crohn’s disease [103]. In this study, Hookworm exposure was assessed by peripheral blood mononuclear cell (PBMC) activation by hookworm antigens in 78 patients with Crohn’s disease and 75 healthy control participants. The change in the proportion of T cells exhibiting CD69 after exposure to crude hookworm antigens was measured, as well as Interferon-ƴ expression by Elispot in front of a panel of six recombinant hookworm antigens. Patients with Crohn’s disease were more often from an urban background compared to controls, while their socioeconomic status was not significantly different. Interferon-γ Elispot responses to hookworm antigens were seen in 36 of 75 controls compared to 20 of 78 Crohn’s disease patients. Multivariate analysis indicated that CD3 CD69 shifts, Elispot reactivity, and place of residence were all independently associated with Crohn’s disease. 

In addition, the authors concluded that the inverse association between Crohn’s disease and hookworm antigen reactivity is consistent with the hygiene hypothesis, but requires further exploration [103]. The “hygiene hypothesis” proposes that the increasing prevalence of allergic and autoimmune diseases in developed countries may be due to the reduction in the incidence of infectious diseases [105]. A “failure to acquire intestinal parasites” is one possible factor that can explain the increasing incidence of Crohn’s disease in developed countries [106]. The strong T helper type 2 (Th2) reaction induced by the helminth parasites neutralized the unbalanced T helper type 1 (Th1)-directed inflammation in the gut in Crohn’s disease [107]. 

In the same manner, the protective effects of hookworm infection against celiac disease were evaluated using Elispot assay [104]. A pilot study of experimental infection with the hookworm *Necator americanus* was undertaken among a group of otherwise healthy people with celiac disease to test the helminth potential to suppress the immunopathology induced by gluten. In a 21-week, double-blinded, placebo-controlled study, the authors explored the effects of *N. americanus* infection in 20 healthy, helminth-naive adults with celiac disease that was well controlled by diet. Staged cutaneous inoculations with 10 and 5 infective 3rd stage hookworm larvae or placebo were performed at week-0 and -12 respectively. At week-20, a five-day oral wheat challenge equivalent to 16 grams of gluten per day was undertaken. Primary outcomes included duodenal Marsh score and quantification of the immunodominant a-gliadin peptide (QE65)-specific systemic interferon-ƴ-producing cells by Elispot pre- and post-wheat challenge. Enteric colonization with hookworm established in all 10 cases, resulting in transiently painful enteritis in 5. Chronic infection was asymptomatic, with no effect on hemoglobin levels. Although some duodenal eosinophilia was apparent, hookworm-infected mucosa retained a healthy appearance. In both groups, wheat challenge caused deterioration in both primary and several secondary outcomes. Experimental *N. americanus* infection proved to be safe and enabled the testing of its effects on a range of measures of the human autoimmune response.

## 6. Mycosis

During the last few decades, a permanent increase in the number of fungal skin or systemic infections has been observed worldwide [108]. Invasive infections caused by *Candida albicans*, a constituent of human amphibiotic microbiota, have been revealed as an increasing problem especially in immunocompromised patients. In this context, the use of the Elispot assay and variations of the technique in mycology started in 1992 with a study of candidiasis, the most frequent mycosis using Elispot assay as a tool for analyses [109]. The authors investigated the stimulation and immortalization of human peripheral blood B lymphocytes specific for *Candida albicans* antigen as a strategy for the generation of human monoclonal antibodies to infectious agents. Subsequently, it was used as a method for the detection of antibody forming cells (human peripheral blood lymphocytes) induced by whole *Candida albicans* cells [110]. In this study, the Elispot technique was developed on plastic wells coated with a purified candidal cell wall mannoprotein (MP) and the method permitted the detection of antibodies directed against MP epitopes shared by *C. albicans* and *C. parapsilosis*. 

The use of Elispot as a tool for the study of immunopathogenesis in a murine model of experimental infection in candidiasis began in 1998 [111]. The increase in frequency of IL-2, IFN-γ-, or IL-4-secreting splenocytes induced by *Candida albicans* mannan and/or monophosphoryl lipid A was detected. These data support the hypothesis that IL-4 is involved in MAN-specific immunoregulatory activities. Ten years later, memory Th17 cells specific for *C. albicans* in human peripheral blood were detected by Elispot [112]. In addition, Th-17 cells were different from Th1, Th2 and Treg cells, because the expression of IL-17, IFN-γ, IL-4 or Foxp3 was restricted to distinct CD4^+^ T subsets. Importantly, stimulation of PBMCs with heated-inactivated *C. albicans* yeast or hyphae induced IL-17 production at the protein and transcriptional levels. These data suggested that memory Th-17 cells are present in healthy individual PBMCs and that some memory Th-17 cells might play an important role in the defense against fungal infections such as *C. albicans*. 

In candidiasis, Elispot has been also used for the study of immunogenic epitopes of secretory aspartyl proteinase 2 (Sap2) [113,114]. Sap2 is the most abundant virulence factor expressed during *Candida* infection, and the principal protein known to induce antibody response during *Candida* infection in humans. Two HLA-DRB1-restricted peptides (p17 and p31) are located in areas of prediction and induced clonal proliferation of IL-2- and IFN-γ-producing lymphocytes by Elispot. *Candida albicans* is one of the most important opportunistic dimorphic fungi responsible for hospital-acquired fungal infection in humans. Candida infection rarely occurs in healthy individuals but it is frequently associated with patients who suffer from acquired immunodeficiency syndromes. To date, there is no effective vaccine against this fungal infection. The authors suggested that S2 might be a potential candidate for vaccine development against *C. albicans* infection or to be utilized as an adjuvant to stimulate the pre-existing CD4 T-cell in other developmental vaccines. 

Several studies have established the potential efficacy of humoral immunity, primarily mannan-specific antibodies, in host protection against *Candida albicans*. Paulovicova et al. (2013) analyzed humoral immune responses induced by immunization with BSA-based conjugates bearing synthetic a-1,6-branched oligomannosides (pentamannosides (M5) or hexamannosides (M6)) mimicking antigenic sequences of Candida cell wall mannan. M6-BSA conjugate induced markedly higher levels of mannan-specific IgG compared to the M5-BSA conjugate. In contrast to the M5-BSA conjugate, the M6-BSA conjugate induced immunoglobulin isotype class switch from IgM to IgG, as revealed also from Elispot analysis [115].

In 2014, a triple FluoroSpot detecting the hallmark cytokines of Th1 (IFN-γ), Th17 (IL-17A) and Th22 (IL-22) was developed and evaluated using human peripheral blood mononuclear cells from healthy donors incubated with either tetanus toxoid, *Candida albicans* extract, mycobacterial purified protein derivative or medium only [116]. The response to *C. albicans* contrasted in that higher proportions of IL-17A single secreting as well as co-secreting cells, in particular, IL-17A/IL-22, were found. The FluoroSpot analysis correlated well with single cytokine Elispot assays run in parallel, and the methods displayed a comparable sensitivity. The results demonstrated the functionality of the FluoroSpot assay for the simultaneous analysis of distinct Th1, Th17, Th22 as well as intermediate cell populations. The method provides a mean for a simple and rapid analysis of the involvement of these cells in immunity and disease [116]. Elispot is a sensitive, robust and versatile assay that can be applied to many different analytes, although it is limited in that it is restricted to the detection of a single cytokine. Dual color Elispot—utilizing two different enzymes that generate substrate products of different colors—has been used, but the results can be ambiguous if one of the precipitating substrate products obscures the other. The FluoroSpot assay overcomes this limitation by utilizing fluorophores for the detection of multiple cytokines and also facilitates analysis of more than two cytokines. Uing selective filters for excitation and emission, fluorescent signals in FluoroSpot can be cleanly separated and individual images of each fluorophore captured, void of interference and bleed-through artifacts. Individual analysis of each analyte is therefore possible, much like a series of separate single color Elispot assays. Double- and triple-stained spots are then identified based on the spot positions in the different filter images.

HIV and *C. albicans* co-infected patients have also been evaluated using Elispot assay. Bauerle et al. (2006) observed an impaired Candida specific T cell repertoire in HIV+ patients that could increase the risk of immune evasion by *C. albicans* [117]. They analyzed oral Candida colonization in correlation to the Candida-specific Tcell response measured by IFN-γ Elispot using different *Candida (C.) albicans* strains. Candida was isolated from 16 of 46 patients, different to the control group, with negative isolation for all subjects. A significant association of higher CD4 cell numbers with both detection of Candida specific T-cells and a lack of oral Candida colonization was observed, but there was no significant correlation between oral Candida colonization and the detection of circulating Candida specific T-cells, viral load or antiretroviral therapy. Thus, local mucosal immunity seems to be more important in the pathogenesis of *Candida* spp colonization than are circulating Candida specific T-cells. In the same year, Burgess et al. (2006) evaluated the impact of antiretroviral therapy and measured cells secreting IFN-γ. They observed that IFN-γ responses to Candida had recovered slowly or remained low in immunodeficient HIV patients responding to antiretroviral therapy [118].

In the context of cryptoccocosis, antigen-specific antibody-forming cells were detected in bronchoalveolar lavage fluid of patients with summer-type hypersensitivity pneumonitis using the method of Elispot [119]. Fifteen years later, Elispot was used to detect cryptococcal meningitis in HIV patients presenting immune restoration disease [120]. The patients presented peaks in the proportion of activated T-cells and IFN-γ secreting cells, and immune restoration disease did not reflect a paucity of regulatory CD4-T cells. 

A unique study on sporotrichosis immunopathogenesis using Elispot to detect cytokines expressing cells in active lesions of patients was developed recently by Morgado et al. (2016). In that study, the detection of IL-10 and IFN-γ expressing cells in peripheral blood elucidated two important features of the parasite-host relationship in sporotrichosis [121]. First, regardless of the clinical cutaneous form, there were no differences in the systemic cytokines expression, suggesting the importance of immunological compartmentalization in this mycosis. The lesion severity was related to more intense IL-10 and NOS2 expression and higher fungal load in the skin. The higher in situ IL-10 expression could be related to regulatory mechanisms for compensating tissue injury, yet favoring fungal persistence in the lesions. Second, on the fungal agent side, the *S. schenckii* antigen was able to induce the expression of IL-10 by PBMC in all patient groups evaluated, including the healthy ones, suggesting it might be a mechanism of immune escape.

There is one more study on the immunopathogenesis of mycoses based on the detection of cytokine secreting cells. Potenza et al. (2016) evaluated Mucorales-specific T-cells producing IFN-ƴ, IL-10 and IL-4 in peripheral blood samples of patients with invasive mucormycosis. They suggested that Mucorales-specific T cells polarized to the production of T helper type 2 cytokines are associated with proven IM and may be evaluated as a surrogate diagnostic marker for IM [122].

## 7. Elispot as an Important Tool in Vaccine, Allergy and Adverse Reactions to Drugs

Elispot may also be employed in other research areas where knowledge of the type and intensity of the immune response involved is necessary and areas that are closely linked to infectious diseases. Two examples have been well documented in the literature: (1) Vaccine development and (2) Detection of allergy.

Elispot has been employed to detect immune response induced by vaccine candidates since cell-based immunity is often pointed out as protective in several infectious diseases. Nowadays there is consensus that a successful vaccine often requires cytotoxic T cells/and a T-B lymphocyte cooperation [123]. The efficacy of vaccine candidates has been accessed by Elispot in different infectious diseases such as Chagas disease [124], influenza [125,126], smallpox [127]; Ebola [128], malaria [129,130], HIV [131], tuberculosis [132,133], among others. All these studies have stressed the importance of dissecting the immune response stimulated by vaccine candidates as a way of defining the protective epitopes, thereby facilitating the design of vaccines based on the construction of chimeras as well as on the detection of peptides which are naturally recognized in the population exposed/protected against an infectious agent.

The literature has shown the importance of Elispot for quickly detecting the immune response to allergens such as trees, grasses, fungi, weeds, dust mites, pollen, food, and dyes [134,135,136,137]. Particularly, regarding medication, the literature has shown the presence of immune responses against different classes of drugs in patients presenting Hypersensibility Syndrome, Drug Reaction with eosinophilia and systemic symptoms (DRESS syndrome) [138], Stevens-Johnson Syndrome [139] (Fu et al. 2012) or similarly under the use of sulfasalazine [140], cephalosporine [141], amikacin [138], B-lactams [142], and allopurinol [143], among others. The fast detection of adverse reactions to the drugs used in the treatment of infectious diseases is critical, and results already published indicate that Elispot is an effective tool for the early identification of these reactions as well as for testing possible immune responses against other medicines available, thus allowing the fast change/adjustment to treatment of affected patients.

## 8. Concluding Remarks 

Neglected tropical diseases blind, maim, disfigure and debilitate hundreds of millions of people in urban slums and in the poorest parts of the world. Since they are widely prevalent, these diseases need to be investigated with cutting-edge approaches. Therefore, Elispot is an excellent tool for obtaining information on the evolution of tropical infectious diseases, on the mechanisms involved in the emergence of different clinical presentations produced by the same pathogen, as well as on the adverse reactions to drugs commonly used in its treatment, and in the testing of new vaccine candidates (summarized in Table 1). These three points are fundamental to the understanding of the interaction between infectious agents and the mammalian immune system. This understanding will improve the clinical management of patients, as well as aid in a more rational development of new therapies and vaccines. 

## Figures and Tables

**Table 1:** Summary of Elispot-based research applied to key tropical disease issues. In the fields of diagnosis and prognosis, even though the use of Elispot is widespread for the study of Dengue, bacterial infections and some mycosis, the diagnostic/prognostic value of Elispot in other diseases such as helminthiasis and protozoan diseases remains to be explored. Despite the fact that Elispot has proven to be a valuable tool for studies of pathogenesis and in the development of vaccines against tropical diseases, it has been little applied to emerging and re-emerging diseases such as Zika, Chikungunya, Yaws and Cholera. Lastly, the epitope identification by different Elispot approaches is present in most diverse scenarios of tropical diseases studies.

	Diagnosis/Prognosis	Pathogenesis	Vaccine development	Epitope identification
Protozoan diseases				
Malaria	-	[12,13,14,25,26]	[15,16,17,18,19,20,21,22]	[23,24]
Toxoplasmosis	-	[27,30]	-	[28,29]
Chagas disease	-	[36]	-	[31,32,33,34,35]
Leishmaniasis	[43]	[42]	[39,41]	[40]
Tropical Arboviroses				
Dengue fever	[45,52,55,56,70]	[47,48,49,50,51,53]	[65,68]	[57,58,59,60,61,62,63,69]
Zika	-	-	-	[72]
Chikungunya	-	[73,74]	-	-
Yellow fever	-	-	[75,76,77]	[77]
Bacterial diseases				
Tuberculosis	[80,81,85,87,89,90]	[83,84]	[83]	[83]
Leprosy	[89,90]	[88]	-	-
Yaws	[91,92]	-	-	-
Cholera	[94]	-	-	[93]
Helminthiasis				
Schistosomiasis	-	[102]	[99,100]	-
Ancylostomiasis	-	[103,104]	-	-
Mycosis				
Candidiasis	[117,118]	[109,110,111,112,116]	[115]	[113,114]
Cryptoccocosis	[119,120]	-	-	-
Sporotrichosis	-	[121]	-	-
Mucormycosis	-	[122]	-	-
